# Role and Significance of Circulating Biomarkers: miRNA and E2F1 mRNA Expression and Their Association with Type-2 Diabetic Complications

**DOI:** 10.1155/2020/6279168

**Published:** 2020-08-20

**Authors:** Mirza Masroor Ali Beg, Amit Kumar Verma, Mohd Saleem, Fayez Saud Alreshidi, Fahaad Alenazi, Hafiz Ahmad, Prakash C. Joshi

**Affiliations:** ^1^Department of Medical Elementology and Toxicology, Jamia Hamdard, New Delhi, India; ^2^Department of Biochemistry, Maulana Azad Medical College, New Delhi, India; ^3^Department of Zoology and Environmental Sciences, GKV, Haridwar, India; ^4^Department of Pathology, College of Medicine, University of Hail, Hail, Saudi Arabia; ^5^Department of Family and Community Medicine, College of Medicine, University of Hail, Hail, Saudi Arabia; ^6^Department of Pharmacology, College of Medicine, University of Hail, Hail, Saudi Arabia; ^7^Department of Medical Microbiology and Immunology, RAK Medical & Health Sciences University, Ras Al Khaimah, UAE

## Abstract

**Background:**

Type 2 diabetes mellitus (T2DM) has emerged as an epidemic affecting more than four hundred million people throughout the world. It is a multifactorial disease with range of environmental and genetic factors responsible for its prevalence. In search of novel biomarkers for recording progress of various metabolic diseases, small noncoding RNA in general and microRNAs (miRNAs) in particular have emerged as the most promising biomarkers for diagnosing variety of diseases including diabetes. An increasing number of studies have been published, reporting the quantification of miRNAs in blood of subjects with diabetes and mostly aimed at identifying miRNA modulation in chronic diabetic complications. Due to its association with immune system homeostasis and potential capability to predict diabetes development, the profile of circulating miRNAs may also provide useful information about diabetes pathogenic mechanisms. Thus, the present study aimed to understand the role and expression of microRNA330 and E2F1 mRNA expression in patients with T2DM. *Methodology*. The present study includes a total of 200 individuals: 100 “individuals with T2DM referred to as “cases” and 100 healthy individuals referred to as “controls”. Extracted RNA was used to synthesise the cDNA for microRNA-330 and E2F1 mRNA expression. Taqman assay method has been used to analyse the microRNA-330 expression in the cases and controls and SYBR green dye was used to study the E2F1 mRNA expression.

**Results:**

Statistically significant difference was observed in all the selected 5 biochemical parameters among T2DM cases and healthy controls. Risk factors like hypertension were observed to be significantly associated with reduced HDL (*p*=0.01), increased TG (*p*=0.0008), and cholesterol (*p* < 0.0001) in hypertensive T2DM cases as compared to nonhypertensive T2DM cases. Obese patients showed significant increase in TG (*p*=0.01) and cholesterol (*p* < 0.0001) as compared to nonobese patients. Similarly, increased TG (*p*=0.001) and cholesterol (*p* < 0.0001) was observed in the case of alcoholic patients as compared to nonalcoholic patients. Also, patients with smoking habit showed increased TG (*p*=0.009*p* = 0.009), cholesterol (*p* < 0.0001), and VLDL (*p*=0.01) as compared to nonsmokers and differences among them was found to be statistically significant. Besides this, significant impact of risk factors like hypertension, obesity, alcoholism, and smoking were observed on microRNA-330 expression and E2F1 mRNA expression. A 7.72-fold increased microRNA-330 and 0.05-fold decreased E2F1 mRNA expression was observed among T2DM cases as compared to healthy controls. Increased expression of microRNA-330 was observed in hypertensive cases (9.61-fold, *p* < 0.0001), obese cases (9.33-fold, *p*=0.0008, alcoholic cases (9.07-fold, *p* < 0.0001), and smoking cases (8.41-fold, *p*=0.01) as compared to nonhypertensive, nonobese nonalcoholic, and nonsmoking cases, and differences among them were found to be significant. Decreased expression of E2F1 mRNA expression was observed in patients with alcoholism (0.03-fold, *p*=0.002) and smoking (0.03fold, *p* < 0.0001) while patients who were nonalcoholic and nonsmokers showed 0.07-fold increase in expression, and differences among them were found to be statistically significant.

**Conclusion:**

The present study demonstrated that increased level of microRNA-330 and decreased level of E2F1 mRNA expression were found to be associated with pathogenesis of T2DM patients. Risk factors such as hypertension, obesity, alcoholism, and smoking may be were found to be associated with microRNA-330 and E2F1 mRNA expressions, and it can prove a reliable biomarker for T2DM disease progression could be linked to chronic diabetic complications.

## 1. Introduction

First mentioned in the Egyptian manuscript about 3000 years ago, diabetes mellitus is one of the oldest diseases known to mankind [1]. Over the millennia, type 2 diabetes mellitus (T2DM) has emerged as an epidemic affecting more than four hundred million people throughout the world. Presently, approximately 415 million people have been affected from diabetes around the world which are likely to increase up to 642 million by 2040 (IDF) [2]. The metabolic disorder is recognized as noninsulin dependent form of diabetes [3] landmarked by hyperglycaemia primarily because of body's inability to either produce insulin or respond to it [4]. It is a multifactorial disease with range of environmental and genetic factors responsible for its prevalence. The causative factors have increased with urbanisation induced sedentary lifestyle,, physical inactivity, and unhealthy eating practices, especially in developing countries. The disease is linked with serious, disabling, long-term complications, including heart related disease, renal disorders, neurological disease, and eye disease, and became one of the major causes of morbidity and mortality globally [5].

In order to plan adequate prevention of T2DM, the evaluation of traditional biomarkers primarily small noncoding RNA in general and microRNAs (miRNAs) in particular have recently emerged as key regulators of metabolism and metabolic disorders. Growing evidence indicates that microRNAs, which are class of small 20–24 nucleotide extended noncoding RNA, that potentially control the expression of thousands of genes [6], are also involved in the progression of diabetes [7]. MicroRNAs function as translational repressors and important regulators of key cellular processes [8]. These traditional biomarkers in the form of microRNA's, which are released by most cells in the body, reaching blood circulation in a very stable form, may be used to assess cell activity at distance [9]. Furthermore, several circulating microRNAs are reported to be implicated in beta-cell activity, differentiation, and both in normal and disease conditions [10]. Through the evaluation of traditional biomarkers, it is then possible to identify subjects who already show metabolic alterations, such as higher blood glucose levels than normal but not yet high enough to be diagnosed as diabetic, hence facilitating better diagnosis and management of the disease [11].

An increasing number of studies have been published reporting the quantification (mostly through real-time qPCR) of microRNAs in blood (either plasma or serum) of subjects with diabetes mainly with the aim of identifying circulating microRNA modulation in chronic diabetic complications [12]. Increased level of microRNA-330 was observed in a study conducted on gestational diabetic (GD) pregnant patients as compared to the nondiabetic pregnant subjects. Several other studies conducted on microRNA-330-3p expression highlighted that E2F1 (E2F transcription factor 1) is one of the most conducive targets transcript [13,14]. In an animal modelled study, it was highlighted that E2F1/E2F2 compound mutant mice have shown excessive polyuria, hyperglycaemia, and decline in blood insulin levels [15]. Overexpression of E2F1 can stimulate beta-cell proliferation activity. Reduced pancreatic size and increase glucose intolerant due to beta-cell dysfunction has been reported in E2F1−/− mice [16]. Realising the emergence of microRNA as a suitable traditional biomarker in diagnosing various metabolic disorders including its expression in relevance to T2DM, the present study aims to evaluate the role of microRNA-330 and E2F1 mRNA regulation in T2DM cases in Indian population primarily to understand the pathogenesis in newly diagnosed type 2 diabetes mellitus patients.

## 2. Materials and Methods

### 2.1. Patient Selection and Sample Collection

The present study was ethically approved (14/08/2015/GKV/IEC/2015) and conducted at institutional ethical committee's Gurukul Kangri University, Haridwar, India. The study was conducted between 2015 and 2019, and all the criteria were followed such as fasting glucose (fasting plasma glucose level of 126 mg/dL or higher) and postprandial glucose (2 h plasma glucose level of 200 mg/dL) were monitored for diagnosis of T2DM disease. Totally 200 study subjects were included in present study: 100 were T2DM patients, and 100 were healthy controls. Written informed consent from all the participants was obtained, and further, 3 ml of peripheral blood sample was collected from all the T2DM cases and healthy control in plain vials and 1 ml in fluoride vials for blood sugar. The samples collected in plain vials were immediately centrifuged at 1500 rpm after collection and stored at -80 °C for further biochemical analysis and cell-free total RNA extraction experiments. A sample of T2DM patients along with healthy controls having BMI >25 kg/m^2^ (obese) and blood pressure ≥140/90 mmHg (hypertensive) were included for the study.

Stored serum were thawed at 4^O^C, and total RNA from serum was extracted by Trizol reagent using recommended protocol (Invitrogen) and then stored at −80 °C until further processing.

100 ng from total extracted RNA was used for Polyadenylation and cDNA synthesis using advanced microRNA cDNA Synthesis Kit (TaqMan, Thermo Scientific) by the following manufacturer protocol. Reverse transcriptase enzyme and other essential reagents were added subsequently for cDNA synthesis to switch in poly (*A*) tailed microRNAs to cDNA using universal RT primer supplied with the manufacturer kit.

From the total extracted RNA, 100 mg RNA was used to synthesise the cDNA using verso cDNA synthesis kit (Thermo Scientific, USA) for E2F1 mRNA expression following manufacturer protocol.

### 2.2. Quantitative Real-Time PCR for miRNA-330 and E2F1 mRNA Expression

Quantitative real-time PCR (qPCR) was performed to quantify the expression of microRNAs-330 by advanced Taqman assay using advanced master mix. SNU6 was used as the internal control to normalise the expression. The reaction was performed in a 25-*μ*l reaction volume using the following program: 95 °C for 5 minutes, followed by 40 cycles of amplification, denaturation at 95°C for 20 seconds, annealing at 60°C for 30 seconds, and elongation at 72°C for 30 seconds. E2F1 mRNA expression was done using SYBR green dye, and GAPDH was used as the internal control to analyse the expression, and the following program was used for amplification: 94°C for 10 minutes, followed by 40 amplification cycles of denaturation at 94°C for 40 seconds, annealing at 58°C for 40 seconds, and elongation at 72°C for 40 seconds. At the end, a melting curve was generated by programming fluorescent measurements every 1°C from 35°C until 95°C to ensure a single PCR product.

### 2.3. Statistical Analysis

All the statistical analysis was done using Graph Pad Prism version 6.05. QRT-PCR data were analysed by the relative cycle threshold (Ct) method, and every sample was examined in triplicate. MicroRNA and mRNA expression levels were calculated by the relative quantification method using 2^–(ΔΔCt)^. The Mann-Whitney U test and Student *t*-test were conducted to observe the significant differences among groups. Results with more than or less than 1 were taken to indicate upregulation or downregulation of microRNA and mRNA expressions. All values have been presented as mean and standard deviation values with *p* value of <0.05 to be considered as statistically significant.

## 3. Results

The entire demographic characteristics have been mentioned in Table 1. In brief 57% male and 43% female T2DM cases were included while control subjects were 55% male and 45% female. T2DM cases with ≤50 years were 60% while 40% were in age group of >50 years. In controls, 50% were in ≤50 years of age group while 50% were in >50 years of age group (Table 1).

### 3.1. Biochemical Parameters among T2DM Cases and Healthy Controls

Biochemical parameters were recorded in T2DM cases as well as healthy controls, and a comparison was drawn for each selected parameter. The difference among them was found to be significant (Table 2). Higher level of uric acid (*p* < 0.0001), total bilirubin (*p*=0.0006), LDL (*p* < 0.0001), TG (*p* < 0.0001), cholesterol (*p*=0.02), VLDL (*p* < 0.0001), blood sugar fasting (*p* < 0.0001), and post-prandial sugar (*p* < 0.0001) was observed in T2DM cases compared to healthy control while lower level of HDL was observed in T2DM cases compared to healthy controls (*p*=0.02).

### 3.2. Association of Biochemical Parameters with Risk Factors

Various biochemical parameters were found to be associated with risk factors of the T2DM. For the present study, 5 biochemical parameters, i.e., HDL (high-density lipoprotein), LDL (low-density lipoprotein), TG (triglycerides), cholesterol, VLDL (very-low-density lipoprotein) have been compared against four risk factors, i.e., hypertension, obesity, alcoholism, and smoking. Impact of these four risk factors (hypertension, obesity, alcoholism, and smoking) has been assessed on each of the five given biochemical parameters. The observed association between them has been observed and evaluated using Mann Whitney *U* test and Student's *t*-test as shown in (Table 3). Risk factor of hypertension was found to be associated with HDL, TG, and cholesterol among T2DM cases. Patients who were hypertensive showed low HDL (30.13 ± 4.17) level while patients without hypertension showed high HDL(32.44 ± 5.65) level, and differences among them was found to be significant with *p* value of 0.01. Other parameters such as TG was 208.3 ± 47.65 (*p*=0.0008), cholesterol was 230.3 ± 43.84 (*p* < 0.0001) among T2DM cases with hypertension, while nonhypertensive patients had lower TG (177.6 ± 42.15) as well as cholesterol (194.8 ± 38.87), and differences among them was found to be significant.

Level of cholesterol among obese patients was higher (258.0 ± 47.45), while in nonobese patients had lower level (192.9 ± 23.52) comparatively, and difference among them was found to be significant (*p* < 0.0001). T2DM patients who were obese were also observed to have higher level of TG (209.8 ± 47.14) while nonobese patients had low level of TG (178.9 ± 47.02), and difference among them was found to be significant (*p*=0.001). Level of cholesterol among obese patients were observed to be high (243.7 ± 43.32) while nonobese patients had lower cholesterol (181.4 ± 14.37) levels, and the difference among them was found to be significant (*p* < 0.0001). Similarly, two of the other risk factors like alcohol consumption and smoking also reflected significant association with T2DM. T2DM patients who were alcoholic had higher level of TG (213.0 ± 51.01) while nonobese had lower level of TG (186.6 ± 46.32), and difference among them was found to be significant (*p*=0.01). Further, patients who had habit of smoking showed higher TG level (205.3 ± 51.42) compared to nonsmokers (183.8 ± 16.17), and differences among them were found to be significant (*p*=0.009). Level of cholesterol among smokers was higher (233.4 ± 47.25) while nonsmokers showed lower level of cholesterol (183.8 ± 16.17), and differences among them was found to be significant (*p* < 0.0001). Higher level of VLDL was found in smokers (36.21 ± 5.43) while low level in nonsmokers (33.41 ± 5.06), and differences among them was found to be significant (*p*=0.01).

### 3.3. MicroRNA-330 Expression and Risk Factors

The study overall exhibited 7.72 mean fold increase in expression of microRNA-330 in T2DM patients as compared to controls (Table 4). Association of risk factors, i.e., hypertension, obesity, alcoholism, and smoking, was observed with microRNA-330 in T2DM cases. Patients who were hypertensive showed a 9.61-fold increase in microRNA-330 expression while nonhypertensive patients showed a 5.40-fold increase in microRNA-330 expression and differences among them were found to be significant with *p* value of <0.0001. A 9.33-fold-increased microRNA-330 was observed in obese patients while nonobese patients showed 6.88 mean fold, and differences were observed to be significant (*p*=0.0008). The impact of alcoholism was also seen to be profound on the expression of MicroRNA-330 in persons consuming alcohol as compared to nonalcoholics. Increased expression of microRNA-330 was observed in alcoholics (9.07-fold) as compared to nonalcoholics (6.12 fold) T2DM patients (*p* < 0.0001). Smoking showed 8.41-fold-increased microRNA-330 expression while nonsmokers had a 6.53-fold microRNA-330 expression, and differences among them were also observed to be statistically significant (*p*=0.01).

### 3.4. E2F1 mRNA Expression and Risk Factors

The impact of selected risk factors, i.e., hypertension, obesity, alcoholism, and smoking, was also observed on E2F1 mRNA expression (Table 5). Alcoholism and smoking were observed to have profound impact on the downregulation of E2F1 mRNA expression. Patients who were alcoholic revealed only 0.03-fold E2F1 mRNA expression while nonalcoholic patients showed slightly higher 0.07-fold mRNA expression (*p*=0.002), manifesting a decrease in expression of E2F1 mRNA in alcoholic cases. Smoking group of patients also exhibited a decreased expression; it was only 0.03-fold while nonsmokers showed slightly higher expression of E2F1 mRNA with 0.07-fold, and difference among them was found to be significant (*p* < 0.0001).

**Figure 1:**
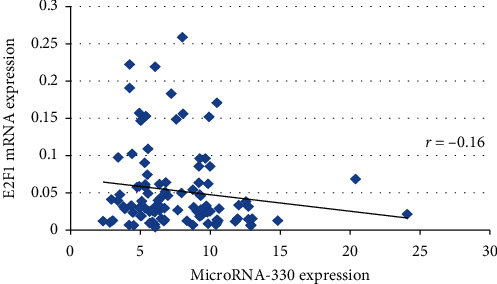
Correlation of microRNA-330 and E2F1 mRNA expression among T2DM cases.

A negative correlation was observed between microRNA-330 and E2F1mRNA expression in T2DM patients. Correlation coefficient with 0.16 (*p* = ns) was observed (Figure 1). These probably suggest that increased microRNA-330 expression may cause the downregulation of E2F1 mRNA expression in the patients.

**Figure 2:**
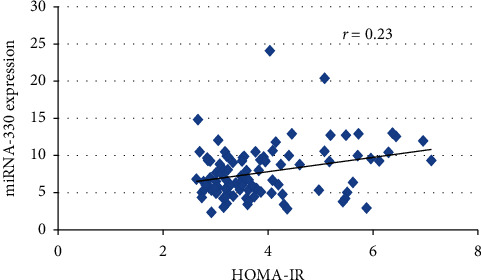
Correlation of HOMA-IR with microRNA-330 expression among T2DM cases.

A positive correlation was observed between HOMA-IR and microRNA-330 expression among T2DM patients. Correlation coefficient with 0.23 (*p*=0.02) was observed (Figure 2). These probably suggest that increase in HOMA-IR will positively regulate the microRNA-330 expression and may cause the increase expression in the T2DM patients.

## 4. Discussion

With more than 400 million patients, type 2 diabetes mellitus (T2DM) is one of the most frequently diagnosed metabolic diseases worldwide. T2DM is associated with genetic, lifestyle, and environmental factors which make tissues insulin-insensitive, resulting in high blood glucose levels. T2DM is also connected with severe long-term complications, including cardiovascular disease, renal failure, neuropathy, blindness, and increase in morbidity and mortality [5]. Significant differences were observed in biochemical parameters, which have the potency to discriminate the T2DM cases with healthy controls. Multiple risk factors such as hypertension, obesity, alcoholism, and smoking were observed to impact the biochemical parameters.

A positive microRNA-330 overexpression was observed for cases with diabetes risk factors such as hypertension, obesity, alcoholism, and smoking as compared to those who were nonhypertensive, nonobese, nonalcoholic, and nonsmokers, who manifested comparatively lower expression. E2F1-decreased expression was linked with obesity, alcoholism, and smoking while nonobese, nonalcoholic, and nonsmokers had slightly higher expression comparatively. A negative correlation was observed among microRNA-330 and E2F1 mRNA expression among T2DM patients with a nonsignificant *p* value of 0.16. Increased levels of microRNA-330-3p can be related to reduction in E2F1 expression potentially and may cause impairment in proliferation of beta cell along with insulin production. It has been shown earlier that cell cycle-related genes involvement is essential to beta-cell compensation [17,18]. A study by Sebastiani *G* in 2017 demonstrated that upregulated microRNA-330-3p was observed in plasma of gestational diabetes mellitus patients and advised that microRNA-330-3p could be the essential indicator in GDM outcome for therapeutic goal [10].

E2F1 involved in cell proliferation and metabolic process by coordinating cellular response by acting as regulatory switch [19]. Studies on protein retinoblastoma RB1, further sustain a major role in metabolism of E2F1 [20]. It has been shown that E2F1 is critical in controlling liver metabolism, regulates uptake of cholesterol, and promotes lipid biosynthesis through transcriptional regulation by lipogenic enzymes [21]. Increased E2F1 mRNA expression has been observed in diabetic patients liver biopsy samples which correlates with the levels of PCK1, which is concerned to hyperglycaemia development in mice [22] and possibly also in humans [23]. In addition to this, E2F1 is directly involved in gene regulation encoding pyruvate dehydrogenase kinase-4 (PDK4), an important regulator of glucose oxidation and nutrient sensor resulting in restricting mitochondrial glucose oxidation [24]. E2F1 helps to regulate metabolic homeostasis via several roles in multiple metabolic tissues. Studies regarding the retinoblastoma protein RB1 additionally support a major role of E2F1 in cellular metabolism in human [25]. However, the function of E2F transcription factors is not clearly known concerning to pocket proteins in beta cells [26,27].

E2F1 plays a major role in hepatic steatosis. It has been demonstrated that E2F1 essentially involved in hyperlipidemia and hyperglycaemia during resistance to insulin and abnormally increased in type 2 diabetes [28]. A study by Iglesias et al. in 2004 on mice model revealed that the E2F1 transcription factors also have essential involvement in regulating both apoptosis and beta-cell proliferation [15].

## 5. Conclusion

Increased microRNA-330 and decreased E2F1 mRNA expressions were observed to be associated in T2DM patients and were found to be linked with various diabetes risk factors. Increased microRNA-330 and decreased E2F1 mRNA expressions may be associated with pathogenesis, increased risk, and exacerbated disease progression of T2DM cases. These changes in expression of microRNA-330 and E2F1 mRNA are not only reliable biomarkers in the diagnosis of T2DM. MicroRNAs have become the most promising biomarkers for the diagnosis, prognosis, and therapeutic options of a variety of metabolic disorders including T2DM; hence, validation on large T2DM population especially from a developing country like India, with increasing diabetic cases each year, can be of profound significance in adequate treatment and prevention strategies of T2DM.

## Figures and Tables

**Table 1:** Demographic and clinical characteristics of T2DM patients and controls subjects.

Variables	T2DM cases (%)	Controls (%)
Age (in years)		
≤50 years	60 (60%)	50 (50%)
>50 years	40 (40%)	50 (50%)

Gender		
Males	57 (57%)	55 (55%)
Females	43 (43%)	45 (45%)

Hypertension		
Yes	55 (55%)	35 (35%)
No	45 (45%)	65 (65%)

Obesity		
Yes	34 (34%)	15 (15%)
No	66 (66%)	85 (85%)

Alcoholism		
Yes	54 (54%)	60 (60%)
No	46 (46%)	40 (40%)

Smoking		
Yes	63 (63%)	60 (60%)
No	37 (37%)	40 (40%)

**Table 2:** Comparison of biochemical parameters among T2DM cases and healthy controls.

Biochemical parameters	T2DM cases (mean ± SD)	Controls (mean ± SD)	*p* value
Uric acid (mg/dl)	5.44 ± 1.79	4.20 ± 0.74	<0.0001
Total bilirubin (mg/dl)	0.86 ± 0.44	0.62 ± 0.20	0.0006
HDL (mg/dl)	31.29 ± 4.98	56.22 ± 9.64	<0.0001
LDL (mg/dl)	145.9 ± 25.4	113.3 ± 14.10	<0.0001
TG (mg/dl)	195.6 ± 49.33	139.5 ± 24.92	<0.0001
Cholesterol (mg/dl)	215.1 ± 45.53	204.2 ± 19.75	0.02
VLDL (mg/dl)	35.17 ± 5.44	25.32 ± 4.81	<0.0001
Blood sugar fasting (mg/dl)	170.2 ± 37.27	92.75 ± 10.88	<0.0001
Postprandial sugar (mg/dl)	256.7 ± 51.61	126.7 ± 9.58	<0.0001

**Table 3:** Comparison of biochemical parameters with different risk factors among T2DM cases.

Variables	Hypertension		*p* value
	Yes	No	
HDL (mg/dl)	30.13 ± 4.17	32.44 ± 5.65	0.01
LDL (mg/dl)	150.4 ± 27.80	144.1 ± 21.58	0.20
TG (mg/dl)	208.3 ± 47.65	177.6 ± 42.15	0.0008
Cholesterol (mg/dl)	230.3 ± 43.84	194.8 ± 38.87	<0.0001
VLDL (mg/dl)	35.76 ± 4.76	34.42 ± 6.12	0.20

Variables	Obesity		*p* value
	Yes	No	
HDL (mg/dl)	32.44 ± 5.48	30.70 ± 4.63	0.09
LDL (mg/dl)	145.9 ± 25.20	145.9 ± 25.70	0.99
TG (mg/dl)	213.0 ± 51.01	186.6 ± 46.32	0.01
Cholesterol (mg/dl)	258.0 ± 47.45	192.9 ± 23.52	<0.0001
VLDL (mg/dl)	36.0 ± 4.95	34.74 ± 5.67	0.27

Variables	Alcoholism		*p* value
	Yes	No	
HDL (mg/dl)	31.76 ± 4.86	30.74 ± 5.10	0.31
LDL (mg/dl)	143.3 ± 28.50	149.0 ± 21.10	0.26
TG (mg/dl)	209.8 ± 47.14	178.9 ± 47.02	0.001
Cholesterol (mg/dl)	243.7 ± 43.32	181.4 ± 14.37	<0.0001
VLDL (mg/dl)	35.74 ± 4.66	34.50 ± 6.22	0.25

Variables	Smoking		*p* value
	Yes	No	
HDL (mg/dl)	30.83 ± 5.06	32.08 ± 4.81	0.22
LDL (mg/dl)	147.6 ± 27.63	142.9 ± 21.11	0.37
TG (mg/dl)	205.3 ± 51.42	179.1 ± 41.19	0.009
Cholesterol (mg/dl)	233.4 ± 47.25	183.8 ± 16.17	<0.0001
VLDL (mg/dl)	36.21 ± 5.43	33.41 ± 5.06	0.01

**Table 4:** Comparison of microRNA-330 expression with different risk factors among T2D cases.

Risk factors	MicroRNA-330 expression		*p* value
	Yes	No	
Overall expression	7.72 ± 3.50		—
Hypertension	9.61 ± 3.41	5.40 ± 1.62	<0.0001
Obesity	9.33 ± 3.98	6.88 ± 2.92	0.0008
Alcoholism	9.07 ± 3.72	6.12 ± 2.41	<0.0001
Smoking	8.41 ± 3.87	6.53 ± 2.36	0.01

**Table 5:** Comparison of E2F1 mRNA expression with different risk factors among T2DM cases.

Risk factors	E2F1 mRNA expression		*p* value
	Yes	No	
Overall expression	0.05 ± 0.05		—
Hypertension	0.04 ± 0.04	0.06 ± 0.06	0.05
Obesity	0.04 ± 0.04	0.05 ± 0.05	0.09
Alcoholism	0.03 ± 0.03	0.07 ± 0.06	0.002
Smoking	0.03 ± 0.02	0.07 ± 0.05	<0.0001

## Data Availability

We assert that the data used in the study will not be shared with anybody or broadcasted in any public domain, since it is impermissible under the policy instructions of GKV. The metadata, supporting the study outcomes, can be accessed from GKV through proper consent; however, privileged data with restricted open accessibility cede institutional authorization and discretion. Other related information and data can also be retrieved from the authors upon reasonable request with permission of GKV.
